# Protocol for a Randomized Control Trial for Tungiasis Treatment in Homa Bay County, Kenya: Dimeticone versus Sodium Carbonate

**DOI:** 10.3390/mps6010012

**Published:** 2023-01-23

**Authors:** Kana Suzuki, Yasuhiko Kamiya, Chris Smith, Satoshi Kaneko, Asiko Ongaya, Evans Amukoye

**Affiliations:** 1 Department of Global Health, School for Tropical Medicine and Global Health, Nagasaki University, Nagasaki 852-8523, Japan; 2 London School of Hygiene and Tropical Medicine, London E4 7RW, UK; 3 School of Biomedical Sciences, Nagasaki University, Nagasaki 852-8523, Japan; 4 Kenya Medical Research Institute, Nairobi 54840-00200, Kenya

**Keywords:** non-inferiority study, tungiasis, treatment, school children, western Kenya

## Abstract

Tungiasis, a World Health Organization neglected tropical disease, is caused by the female sand flea. Most clinical trials for tungiasis use expensive or impractical drugs, which are difficult for residents to use. However, in western Kenya, communities successfully treat tungiasis with sodium carbonate. We hypothesise that the topical risk-difference of 5% sodium carbonate is no more than 10% non-inferior to dimeticone (NYDA^®^) for tungiasis treatment. This is a protocol for a non-inferiority study, which will be randomised and with an observer-blinded control. The study will have two arms: 5% sodium carbonate and NYDA^®^, one on each foot, and will take place at state primary schools in Homa Bay County, Kenya. Fleas identified among school children aged 8–14 years with sand-flea lesions will be enrolled in the study. For each participant, the viability of the embedded fleas, clinical signs including inflammation, and symptoms will be monitored for seven days after treatment. The proportion of dead fleas will be compared in the primary analysis. All adverse events will be monitored throughout the study period. We expect to identify the most effective treatment between sodium carbonate and NYDA^®^ for tungiasis, which can be adopted in the community.

## 1. Introduction

Tungiasis is a cutaneous parasitosis caused by the female sand flea *Tunga penetrans* [1], which is distributed in tropical and subtropical regions of the world. Tungiasis is prevalent in Africa and is recognised as a neglected tropical disease by the World Health Organization (WHO) and has also been predicted to be a target for future priority measures (Sankei-photo, 2019). *Tunga penetrans* is a unique species within the realm of fleas, in that non-fertilised females penetrate the skin and remain there until they die in situ after 4–6 weeks [2,3]. It is one of a few parasites that can maintain its life cycle within sleeping quarters and is capable of completing its life cycle indoors in dwellings without a solid floor [4]. Its eggs are expelled and fall directly to the floor when an infected person sleeps, or later when the bed is made. When the floor is swept, the eggs may be transferred to crevices and holes that contain sufficient organic matter for larval development. Adults emerging from pupae adhere to and penetrate the skin when a person places their bare feet on the dwelling floor [5].

Its age-specific prevalence shows a characteristic pattern, with a maximum prevalence in the elderly population and children aged 5–14 years [6]. Children with tungiasis are reported to have disproportionately high levels of absenteeism from school and perform worse academically than unaffected pupils, because constant itching and pain make it difficult for them to concentrate on schoolwork [7].

### Treatment Options for Tungiasis

If not managed properly, infestation by *Tunga* fleas, commonly known as jiggers, can result in health and socio-economic effects. The surgical extraction of embedded sand-fleas is common, but has several shortcomings. People living in endemic areas may use safety pins, needles, scissors, knives, or sharpened pieces of wood [8]. It is time-consuming and painful, and often results in infections as well as secondary infections such as tetanus, HIV, and hepatitis B and C. One study in sub-Saharan African children showed that inadequate treatment of jiggers is a source of hepatitis B and C transmission [9,10]. Individuals may also abstain from treatment due to the associated pain. Antibiotics and anti-tetanus toxins must be applied to treat secondary infections after jigger removal, and this is often an unmanageable expense.

The Kenyan guidelines [11] recommend the use of antiseptic solutions, including Savlon, potassium permanganate, and hydrogen peroxide followed by petroleum jelly. Flea repellents include N, N-Diethyl-meta-toluamide, Zazarin (coconut oil), neem and coconut oil preparations, jojoba oil, and aloe vera extract. Silicone oil (dimeticone) is also strongly recommended, since it is a more effective and non-toxic remedy. Trends in the use of therapeutic agents have changed significantly in the last eight years. There is ongoing research into therapeutic agents, compounding methods, and application methods, with a focus on increasing the availability of inexpensive treatments without side effects such as pain and itching. Current studies are focusing on the quality of life for patients and the use of naturally derived treatments. A manual search and literature search of PubMed and MEDLINE (EBSCOhost) without language restriction resulted in 141 initial references, from which we finally selected nine relevant articles which showed 13 different treatments. Six randomised control trials and three intervention studies have studied treatments related to tungiasis. Studies are being carried out mainly in South America and East Africa.

#### Locally Available Treatments

Most clinical trials for tungiasis have studied expensive or impractical drugs such as ivermectin, metrifonate, and dimeticone, which are difficult for residents to use on a sustainable basis. In Kenya, however, communities have been successfully treating tungiasis with a herbal-formula lotion composed of neem seed and coconut oils [12]. A recent study conducted in coastal Kenyaby Elson et al. (2019) showed that although 20% neem plus coconut oils did not kill more fleas than potassium permanganate within seven days, it resulted in better secondary outcomes. Specifically, the acute symptoms were much reduced and children had higher odds of being pain-free. Another interventional study [13] was conducted in Muranga County, using 100% coconut oil. However, due to the short observation period of that study, it was unclear whether the fleas died according to their natural lifecycle or due to the intervention. The aforementioned are common local treatments in eastern Kenya, but not in western Kenya, which is far from the ocean. In western Kenya, a local practice is to use sodium carbonate, locally referred to as Magadi or Magadi soda. This naturally available chemical is used in industries for manufacturing glass, cake glazing, as an anti-caking agent for pastries, and within chemical detergents applied for various purposes in homes. Kenyan communities use Magadi to soften green vegetables and meat during cooking. It sells on the local market at approximately KES 10 (USD 0.08) for 50 g. A study conducted in 2016 demonstrated its effectiveness in Siaya County, Kenya [14] and was published after the National Policy Guideline of recommended treatments was developed in 2014. The study of sodium carbonate did not perform practical comparisons, but concluded that it has some degree of effectiveness as a treatment. There is a need to conduct a comparative study prior to including sodium carbonate within the government-recommended treatments. 

Dimeticone is strongly recommended by the WHO for the treatment of tungiasis. A study conducted in Kenya showed that dimeticone applied to the feet up to the ankles killed 78% of embedded sand-fleas within seven days [15]. At an informal meeting of the WHO/Pan American Health Organization in 2021 experts recommended dimeticone as the standard treatment for tungiasis, and it is the only topical treatment that is indicated on the WHO tungiasis website. In addition, the previous review article also concluded that there was sufficient evidence for the dimeticone-based product [16], and increased its availability in Kenyan health facilities. However, it is expensive and not available elsewhere in the local area. 

Sodium carbonate is said to emulsify and remove fat and secretions on the surface of the skin [17]. It has long been used in food preparation and medicine and is safe for humans. In Tanzania, sodium carbonate contaminated with fluoride has been reported to cause fluorosis in human teeth, for instance, when used to tenderise meat [18]. Trona is mined in Kenya, is the source of almost pure sodium carbonate, as well as Magadi soda, and is not contaminated with fluoride [14]. Inhalation of sodium carbonate can lead to adverse effects such as respiratory-tract irritation, cough, shortness of breath, and pulmonary oedema. When using sodium carbonate, care must be taken to avoid ingestion or inhalation of the powder, and to allow for the free flow of fresh air. Accidental or intentional overexposure to sodium carbonate may occur via the oral route. However, a study of acute oral toxicity did not report high toxicity, due to the neutralisation of carbonate in the stomach to bicarbonate and/or carbon dioxide [19]. Contact with eyes can provoke a burning sensation. To avoid these potential hazards, in this study sodium carbonate will be handled with care, and we will choose open preparation workplaces with good airflow.

NYDA^®^ contains two dimeticones or silicone oils with different viscosities and a high creeping property. In many European countries, it is commercialised as a medicinal product for the treatment of head-lice infestation, and is produced by G. Pohl-Boskamp GmbH & Co. KG, Hohenlockstedt, Germany [20,21]. The extremely low viscosity (100 cSt) of NYDA^®^ enables it to penetrate the trachea (respiratory airways) and thereby suffocate the embedded insect. Dimeticones are safe, since they are biochemically inert, non-toxic, non-carcinogenic, and non-teratogenic [22,23]. Due to the physical mode of action of dimeticones, it is highly unlikely that sand fleas would develop resistance. The only disadvantage of the agents is that they are inflammable; thus, it is recommended that individuals should stay away from fires for up to 2 h after application [24].

This is the research protocol: in this study, we compare experimental (dimeticone) and local (sodium carbonate) treatments, aiming to assess the non-inferiority of local treatments of tungiasis among children, who are the key target-population of this disease. In addition, we will investigate further treatment strategies that apply to the local area.

**Hypothesis:**  *In this study, we hypothesise that the topical risk-difference of 5% sodium carbonate is no more than 10% non-inferior to dimeticone (NYDA^®^) when used in the treatment of tungiasis.*

**Figure 1:**
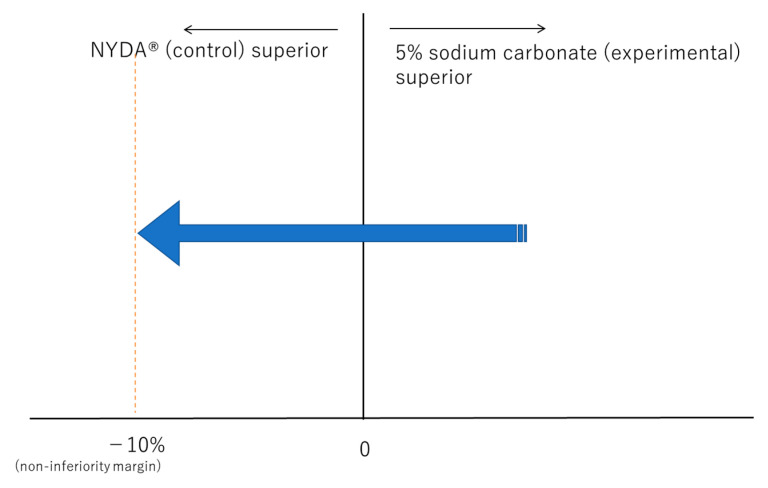
Non-inferiority margin and the relationship with 95% confidence interval.

Figure 1 shows the non-inferiority margin and the relationship with 95% confidence interval (95% CI) of the difference between experimental and control groups. If the risk difference of cure rate (95%CI) is greater than −10%, it can be shown than 5% sodium carbonate is not inferior to NYDA^®^.

## 2. Experimental Design

### 2.1. Objectives

Main objective: To compare 5% sodium carbonate and dimeticone in the treatment of tungiasis among children in Kenya.

Specific objectives:

(1) To compare the cure rates of the two treatments (5% sodium carbonate and dimeticone) within seven days.
(2) To assess the safety of the two treatments by determining the incidence of adverse effects.
(3) To compare the acute pathology scores of the two treatments.
(4) To assess the acceptability of the two treatments in the community.

### 2.2. Justification

**Figure 2:**
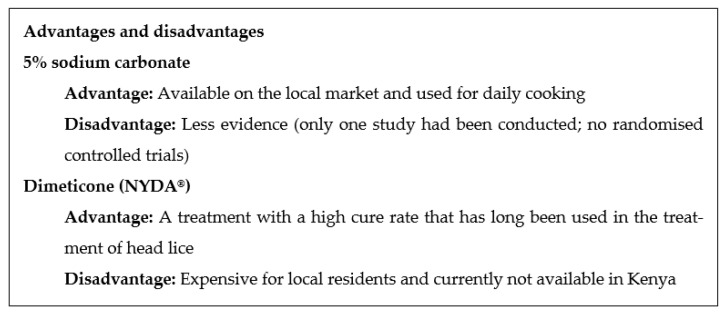
Advantages and disadvantages of the treatments.

In this study, we will compare two treatments for which effectivities have been evaluated and studied in Kenya. Sodium carbonate is available in the local market, whereas NYDA^®^ (a mixture of two dimeticone oils with different viscosities and spreadability) is not yet available in the Kenyan market. The advantages and disadvantages are shown in Figure 2. In terms of mechanism of action, sodium carbonate can soften the tissue and push fleas out by allowing chemicals to enter the body of the flea, due to changes in osmolarity and swelling [14], while dimeticone can penetrate the abdominal cone of fleas, killing them. According to a previous proof-of-principle study [15] the mechanism of action of dimeticone is purely physical. The substance occludes the tracheae of *T. penetrans*, resulting in parasite death.

Solutions of sodium carbonate, typically containing 1% water, are used in personal skin-care products. Application to the feet results in an exposed surface area of 1120 cm^2^ (TGD, Part I, Annex VI). Considering a film thickness of 100 µm and a percutaneous absorption of 0.1%, the amount of sodium carbonate absorbed via the skin would be 0.1 mg. Assuming an exposure time of 20 min, this would amount to 0.0014 mg per application. The estimated systemic dose of sodium carbonate would be equal to 0.0014/60 = 2.3 × 10^−5^ mg/kg body weight [19]. Therefore, the exposure to sodium carbonate via the method of soaking a foot in a 0.5% sodium carbonate solution for 15 min, which is planned for this study, can be regarded as negligible.

### 2.3. Study Design

**Figure 3:**
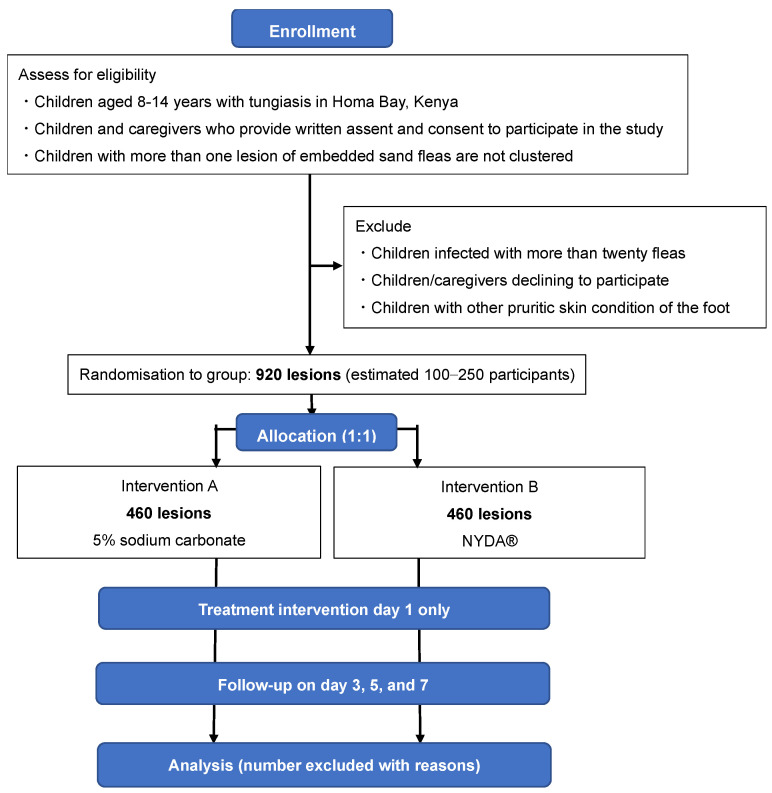
Flowchart of the study.

The study will utilise a randomised design, compared to standard treatment intervention. Figure 3 shows the flowchart of this study. It will have two arms: treatment of embedded sand-flea infestations with 5% sodium carbonate, versus dimeticone. Randomisation will be performed according to treatment. A non-inferiority design is chosen, since it would be unethical to withhold an existing treatment that has demonstrated efficacy. This study will not measure any environmental intervention; therefore, we can measure only efficacy, not effectiveness. All participants will receive both treatments, one on each foot. Therefore, it is not possible to blind participants and interveners to treatment-group assignments. The study will be conducted during the dry season, when the prevalence of tungiasis is known to be at its peak, and at a time not impacted by holidays.

### 2.4. Study Area

The study will be conducted at state primary schools in Homa Bay County, Kenya. The study county has eight sub-counties, among which Ndhiwa and Suba South have been chosen for this study, due to the high prevalence (1.1%) of tungiasis in those areas.

### 2.5. Study Population 

The study will recruit children aged 8 to 14 years. Children will be included if diagnosed with *Tunga penetrans* infestation and manifesting symptoms and signs characteristic of tungiasis and classified as Fortaleza stage II or III [2].

#### 2.5.1. Eligibility Criteria

1. Children aged 8 to 14 years who have been infected with tungiasis in Homa Bay, Kenya (the most commonly affected age-group, and who can reliably answer questions).
2. Children and caregivers who provide written assent and consent to participate in the study.
3. Children who have more than one lesion of embedded sand-fleas that are independent (not clustered) and easily observable, on both feet.

#### 2.5.2. Exclusion Criteria

1. Children infected with more than twenty fleas. These are defined as severe cases [25] and need to be immediately referred to the nearest health facility for treatment or to the medical doctor cooperating in this research, for consultation.
2. Children/caregivers who decline to participate.
3. Children with other pruritic skin-conditions on the foot.

### 2.6. Outcome Measures

The primary endpoint is the cure rate on day seven, following treatment with either 5% sodium carbonate solution or dimeticone. Embedded fleas will be assessed using a digital handheld microscope at the baseline, and on days 3, 5, and 7 for four signs of viability: expulsion of eggs, excretion of faecal thread, excretion of faecal liquid, and pulsations/contractions. An embedded flea will be considered non-viable if none of the four signs of viability is detected during 15 min of observation.

Secondary endpoints are adverse effects (nausea/vomiting, arthralgia, fever, headache, fatigue/malaise, myalgia, and chills), acute pathology scores (assessing pain, itching, erythema, warmness, oedema, desquamation, fissure, suppuration, ulcer, abscess, and lesions in clusters), and differences in feeling and ease of children during the 7-day period after the treatments. Each participant will also be asked to rate their experience of pain and itching using a quick help-sheet with illustrations. An inflammation score [15] will be used for this assessment.

### 2.7. Non-Inferiority Margin Settings

Initially, we would apply the fixed-margin method recommended by the Food and Drug Administration; however, there is insufficient data to estimate the risk ratio of standard intervention [26]. The European Medicines Agency [27] has indicated that “In such situations, it will be difficult to specify delta using the considerations outlined above; however, the best efforts should still be made to produce an indirect confidence interval for the new product against placebo using whatever data exist for the reference.” From this regulator’s view, it is best to consider delta somehow by using indirect data when studying treatments with no evidence of comparison to a placebo. Therefore, we decided to apply a method of determining margins based on scientific theory.

The risk ratio is superior to the risk difference in terms of robustness to differences in rates of outcome between previous studies and non-inferiority studies [28]. In addition, the risk difference is considered an index when analysing binary categorical data. Since the risk ratio is commonly used when performing a time-to-event analysis, the non-inferiority margin was set, based on the risk ratio.

1. Estimating the risk ratio of standard intervention:

Since there is no placebo comparison of either 5% sodium carbonate or dimeticone, we will estimate whether the treatment group shows the same results as those reported by [29].

2. Setting the risk difference of standard intervention using the lower limit of the effect estimate:

95%CI: −0.563 to −0.299.

3. Setting the retention rate and calculating the non-inferiority margin (Wangge et al. [30]):

1 − ε = 0.67

Δ = (1 − 0.67) × −0.299 = −0.09867.

4. Range of interpretation: ±10%.

### 2.8. Sample-Size Calculation

Based on the hypothesis, there is a difference between treatment with 5% sodium carbonate, with a 70% cure rate [14], and dimeticone, with a 78% cure rate [15]. Although it depends on the result of the pilot study, we would like to calculate the sample size assuming that both cure rates are 80%. The sample size was calculated using the method proposed by Schoenfeld [31], assuming a non-inferiority margin of 10% (δ = 0.1) and that the true mean cure-rates of the treatment agent (sodium carbonate) and the active control (NYDA^®^) are 80% (pT = 0.80) and 80% (pC = 0.80), respectively. We calculate the optimum sample needed to achieve 90% power (1 − β = 0.9) at the 5% level of significance (α = 0.05) with equal allocation (*k* = 1) for demonstrating non-inferiority for the clinical-cure assessment of efficacy. Since one to five lesions per foot will be studied per participant, the intra-class correlation coefficient (ICC) (1, *k*) with *k* = 2–5, would be used to evaluate the intra-rater reliability of the values, yielding an estimated ICC = 0.5. As the cluster size is two, the design effect is calculated as 1 + (2 − 1) × 0.5 = 1.5. A total of 920 (460 per arm) lesions will be recruited, considering a 10% rate of loss to follow-up. 

Before conducting this non-inferiority trial, a cohort study will be conducted as a pre-study. The sample size is estimated to have an 80% efficacy rate, absolute precision is 10%, and therefore 62 lesions are enrolled in each concurrent, parallel-treatment cohort.

## 3. Procedure

### 3.1. Sampling and Recruitment

A multi-stage sampling method will be used in this study. Stage 1 will involve the sampling of sub-counties, stage 2 will involve the sampling of schools, participant selection will be performed in stage 3, and stage 4 will involve the sampling of embedded sand-fleas.

Stage 1: Sampling of sub-counties:

Ndhiwa and Suba South sub-counties will be sampled because they have the highest number of reported cases of tungiasis in Homa Bay County. 

Stage 2: Sampling of schools:

Primary schools in the study area are ranked according to the number of reports made to the Health Department. Therefore, we will visit schools in areas with the highest number of reported cases and with the highest number of affected students, in order. 

Stage 3: Sampling of participants:

In each school, children aged 8 to 14 years will be selected in descending order of grade. At the beginning of the study, a sealed envelope will be used to randomise the two treatments. In the sealed envelopes will be written “Right foot: dimeticone, Left foot: 5% sodium carbonate” or “Right foot: 5% sodium carbonate, Left foot: dimeticone” with a participant number. Before randomisation, lesions will be sampled to avoid selection bias.

Children will be informed not to manipulate the lesions during the next 7 days. Children whose skin conditions are suspected to be due to tungiasis by teachers, caregivers, and healthcare workers (including community health-volunteers) will be screened for eligibility to participate in this study. Such children and their caregivers will then be contacted by the local co-investigator and their field assistants to ask if they are willing to participate in the study, after explaining the study objectives and procedures. Informed consent from caregivers and assent from children will be obtained after both agree to participate. Participants will be randomly selected until the point of saturation is reached.

Stage 4: Sampling of embedded sand-fleas:

We will sample participants with a smaller number of lesions sides, and select one to five embedded sand-fleas per foot. Because it is difficult to distinguish the characteristics of individual lesions [15], we will limit the number of lesions studied. The growth level of the target lesions should be stage II or III, according to the Fortaleza classification [2]. Only lesions that can be clearly distinguished from one another with viable embedded sand-fleas will be included in the study. Lesions will be chosen such that their location will make it possible to use a handheld digital microscope. Each lesion will be photographed and circled with a marker, and its location and stage noted in the patient’s record.

### 3.2. Randomisation, Allocation, and Blinding

All study participants will receive 5% sodium carbonate and dimeticone, with one treatment per foot. Randomisation will be carried out at the treatment level and not the individual level. Trained nurses will perform the intervention. It is not possible to blind the intervener administering the treatment because 5% sodium carbonate will require soaking the foot, whereas NYDA^®^ will be applied directly to each lesion. As the two treatments will not change the skin colour of the feet, the principal investigator will be a blinded observer.

### 3.3. Recruitment Strategy

Children deemed eligible for the study will be recruited at school. A trained research assistant will briefly explain the research and after obtaining informed consent, the research assistant will visit the home with the child. The assistant will meet the caregiver of the child at home and ask if they can set up a meeting to explain the study. If the caregiver agrees, they will be given an informed-consent sheet and the research assistant will explain the subject of the investigation to the participating children and their caregivers using the explanatory documents, and will obtain their consent using the study consent form. The documents are written in the local language and English. They will be given time to ask questions for clarification before signing the informed-consent form and assent form (for children aged 13 years and older), and will be informed of their right to withdraw at any point.

There will be a health talk on tungiasis management and prevention for the community and schools before starting the study. The talk will be given through the Japan International Cooperation Agency partnership programme “Sustainable tungiasis control project in Homa Bay County, Kenya”.

According to previous studies, a longer observation period is required to have a better understanding of the outcome; thus, in this study, the primary and secondary observation periods are set to 7 days, including the suppression of new flea-proliferation, because we deduct 20 days (the period until it becomes an adult) from the 4–6 week flea lifespan [2,3,4]. Since the growth level of the target lesions is stage II or III [2], this observation period is considered sufficient. The time of intervention is set as day 1, according to the studies conducted by Ambenje et al. [14] and Nordin et al. [32]. Interventions will be administered after school in primary and secondary schools, in a place where participants feel comfortable answering questions and undergoing treatment applications. Due to the potential hazards of sodium carbonate, a well-ventilated place will be chosen.

A structured questionnaire will be used to collect the data. The questionnaire is written in English, but will be translated into the local language by research assistants. The questionnaire will take 10 to 15 min to complete.

After washing the affected area with soap and running water, both treatments will be applied, one to each foot. One affected foot will be soaked in the 5% sodium carbonate solution for 15 min. The solution will be made by dissolving 50 g of sodium carbonate in a litre of warm potable water within a deep basin. NYDA^®^ will be applied to the affected area of the other foot. In a study that examined the application method using NYDA^®^, targeted application killed embedded fleas more rapidly than when the whole foot was covered [32]. This method of application to the target area will be used in the present study. The reagent will be drawn into a 5 mL syringe to which a flexible tube is affixed. Three drops of NYDA^®^ will be applied to the targeted area. Each drop corresponds to approximately 0.05 mL of NYDA^®^, and the procedure will be repeated three times within 10 min, to ensure that a maximum amount of dimeticone has entered the abdominal cone of the parasite within a short period. This requires approximately 0.15 mL per embedded sand-flea. Clean socks will be provided to cover the foot after treatment application. If the child is wearing sandals, we will provide a pair of socks with the first and second toes separated; if the child is barefoot, we will provide a pair of sandals and socks.

### 3.4. Study Tools

Signs of viability of embedded fleas will be determined using a handheld digital video-microscope with a 3 mega-pixel optical resolution. In addition, clinical photographs should be taken before and after treatment. If a child has another skin disease with tungiasis, we shall keep a record of it, since we will observe a combination of the skin diseases.

The following forms will be printed and used in the field to collect data:

− Questionnaire for study participants: a structured questionnaire;
− Case observation/record form;
− Adverse event/effect record form.

To investigate seasonality associated with the research environment, we will also use a thermometer and hygrometer for assessment.

### 3.5. Data Collection and Storage

Data will not be collected on the names and affiliations of the participants. If they wish to discontinue the interview, observation, and treatment, their decision will be respected without any objections. Participation of school children in this study is totally voluntary. Since this is a school-based study, consent will first be obtained from each school. Informed consent will then be obtained from each participant, in accordance with the Ottawa Statement, an international guideline for cluster-randomised trials. For lesions not studied, dimeticone will be applied later, if desired. The area to be observed will be marked with an oil-based pen and photographed with a smartphone. Video recordings with a smartphone will be made only at the time of intervention, to confirm the intervention method. During the treatment, skin symptoms will be monitored until the final decision on the 7th day, using a checklist. Lesions will be examined and staged according to the Fortaleza classification [2], and counted (live/dead). The cure rate will be calculated at each stage, and significant differences at each stage will be tested. Participants will be observed and queried about adverse effects, and the severity score will be used to assess symptoms of acute tungiasis, including pain and itching. Feeling and ease of each treatment will be assessed using a questionnaire. Trained nurses will monitor the participants, and interviews will be conducted under the guidance of a medical doctor at treatment initiation and during the final decision. 

### 3.6. Pre-Study

A cohort study will be conducted as a pre-study to confirm the efficacy rates. The method is the same as in this non-inferiority trial, and 124 (62 in each cohort) lesions will be enrolled in the study.

## 4. Expected Results

Based on the results of previous studies, the treatment of tungiasis is not sustainable for rural communities. In this study, the primary endpoint will be the cure rate on day 7, following treatment with either 5% sodium carbonate solution or dimeticone, while the secondary endpoints will be differences in adverse effects and acute-pathology scores on day 7, as well as differences in feeling and ease on day 7. Therefore, this study can contribute toward designing a locally sustainable management-cycle that will improve the cure rate, reduce the scores of adverse effects and acute pathology, and sustain the acceptable local treatment for children in Kenya. We hope to help adopt new treatments into the national guidelines of Kenya. The treatment will be cheaply available locally and accessible to poor families suffering from tungiasis in this study area.

### 4.1. Statistical Analysis

For the primary outcome, logistic regression analysis (stepwise regression) will be performed, because it is binary. Since all secondary outcomes are score data, they are continuous measures; thus, analysis of covariance will be performed using two variables (sex (male or female) and footwear use (shoes or sandals)) as covariants. This is because previous studies have shown that the male sex is a risk factor for tungiasis, and that the thickness of the skin of the foot may change depending on whether sandals are used, which may affect the penetration of the treatment [32]. We will use generalized estimating equations for side effects such as pain and itching, to consider relative risks. The per-protocol set, which analyses only subjects who adhere to treatment, is more likely to show superiority or a significant increase in risk, while the intention-to-treat analysis is more likely to show non-inferiority with or without significant differences. Therefore, a per-protocol approach will be adopted for this non-inferiority study. All data will be analysed using EZR version 1.60 (Jichi Medical University Saitama Medical Center, Saitama, Japan). Missing values will be imputed using the multiple-imputation method. For the main analysis, we will use one-sided *p*-values at an *α* = 0.025 level of significance; for others, we will use two-sided *p*-values at an *α* = 0.05 level of significance.

### 4.2. Monitoring Plan for the Study

An independent monitor will oversee the study, apart from the data and safety monitoring committee, which consists of several team members. This is because this study is relatively small-scale, with treatments already locally used, and there is little possibility that significant safety problems will occur. The monitor will confirm the appropriate implementation of the study, proper recording of necessary items, and data reliability. Specifically, the monitor will review the research protocol, informed consent form, questionnaire, records of observations and follow-ups, and observe the drugs to be used. Again, at the study site, the monitor will observe any deviation from the research protocol, protect the rights and safety of the participants, and advise correction if any problems arise. The monitor will also propose a decision to terminate the study when serious adverse effects/events occur or when a significantly superior effect is confirmed in one treatment group by the interim analysis. 

### 4.3. Early Termination of the Study (Stopping Rule)

Due to the difficulty of proper evaluation in the middle of a study and information bias resulting from continuation, we do not consider it necessary to conduct an interim analysis for this short-term, low-risk study.

### 4.4. Adverse Events/Effects

All adverse events, whether serious or non-serious, will be recorded until day 7, which is the end of the study, after the participant and his/her caregivers have provided assent and consent and enrolled in the study. If a participant experiences an adverse event after the informed-assent and consent forms are signed, but the participant has not started to receive the study intervention, the event will be reported as unrelated to the study treatments. A serious adverse event for this study is defined as any untoward medical occurrence that is believed by the investigators to be causally related to the study treatments and results in any of the following: a life-threatening condition with an immediate risk of death, severe or permanent disability, and prolonged hospitalisation. Serious adverse events occurring after a participant discontinues the study will not be reported unless the investigators feel that the event may have been caused by the study drugs or a protocol procedure. The investigators will determine the relatedness of an event to the study treatment, based on a temporal relationship to the study treatment and whether the event is unexpected or unexplained given the participant’s clinical course, previous medical conditions, and concomitant medications. All adverse events will be assessed as part of routine monitoring. Any adverse event that can be attributed to the intervention will be specified as a secondary outcome. However, we cannot know which treatment might cause adverse effects with general symptoms such as fever and high blood-pressure, since all participants will receive both treatments. Therefore, those general symptoms will be checked for the safety of the study and to provide information for a doctor to judge whether to continue the research; other acute pathologies, such as skin symptoms, can be analysed by each treatment.

The study will carefully evaluate the safety and harm of the interventions, specifically, the quality and proper preparation of 5% sodium carbonate and dimeticone, and the procedure for applying these solutions to the participants’ feet. The principal investigator or co-investigators will follow any clinically significant abnormalities until resolution or until a clinically stable endpoint is reached. 

If any participant should react to the study treatment, the study nurses will detect it and take the child to the nearest health facility, a subcounty or county hospital, for appropriate care. 

### 4.5. Hazards and Risks Associated with the Study

If improperly handled, or in the event of accidental exposure, sodium carbonate can pose some hazards to health and safety. If participants experience adverse effects such as irritation, blisters, or burning sensation (which generally happens when a concentrated substance gets into the eyes or is inhaled), we will immediately flush the skin with plenty of water and provide necessary treatments or refer the participant free of charge to the nearest health facility.

Another possible risk is that the child may be teased for having jiggers, since this will be revealed to other children in the school by participation in the study. Before selecting participants, we will provide information on tungiasis through health promotion, so that all children will have proper understanding, to reduce prejudice, discrimination, or stigmatisation toward people affected with the disease. By so doing, we aim to minimise the mental burden on participants. In addition, confidentiality will be maintained throughout the study. Any severe adverse reactions (such as dermatitis, fever, and headache) and events occurring during the follow-up period will be recorded and considered for potential association with the treatment.

In the case that treatment is needed at a doctor’s discretion, participants will be immediately referred to the hospital for treatment, and if the treatments show no effect they will be stopped immediately, before study completion. The treatment will be applied not only to the lesions to be studied but also to other identified lesions. In addition, excluded participants (such as people with embedded sand-fleas on only one foot) will be treated with the agent that works best when the intervention is completed.

### 4.6. Confidentiality

Data will not be collected on the names and affiliations of the participants. We shall keep everything they tell us as confidential as possible. The questionnaire survey will also be conducted in privacy. We will talk with teachers to keep the information confidential. The participant’ personal information will not be mentioned in any reports or publications. 

## Data Availability

Not applicable, as it is a study protocol.
